# Correction: Interprofessional collaboration during a specialised mobile palliative care service pilot in the rural area of Lucerne

**DOI:** 10.1371/journal.pone.0324193

**Published:** 2025-05-12

**Authors:** Sahra Maria Anna Bucher, Anne Marie Schumacher Dimech, Beat Müller, Patrick E. Beeler

Luzern is spelled incorrectly throughout the paper. The correct spelling is Lucerne.

There is an error in the article XML causing the second author’s name to be indexed incorrectly. The name should be indexed as Schumacher Dimech AM. The correct citation is: Bucher SMA, Schumacher Dimech AM, Müller B, Beeler PE (2024) Interprofessional collaboration during a specialised mobile palliative care service pilot in the rural area of Lucerne. PLoS ONE 19(9): e0308256. https://doi.org/10.1371/journal.pone.0308256.

There are errors in the Author Contributions. The correct contributions are:

Conceptualization: Sahra Maria Anna Bucher

Project administration: Patrick E. Beeler

Supervision: Patrick E. Beeler

Validation: Anne Marie Schumacher Dimech

Writing – original draft: Sahra Maria Anna Bucher

Writing – review & editing: Anne Marie Schumacher Dimech, Beat Müller, Patrick E. Beeler
