# Supplementary material for: Interprofessional collaboration during a specialised mobile palliative care service pilot in the rural area of Lucerne
Source: PLoS One. 2024 Sep 18;19(9):e0308256. doi: 10.1371/journal.pone.0308256 (PMC11410264; doi:10.1371/journal.pone.0308256)
Supplement: S1 File — (DOCX) [file pone.0308256.s001.docx]

1. Questionnaire interprofessional collaboartion

| **Demographische Daten** |
| --- |
| Bitte geben Sie Ihre Initialen an. |
| Wie alt sind Sie? |
| Bitte geben Sie Ihr Geschlecht an.   - Männlich - Weiblich - Divers - Möchte ich nicht angeben |
| Bitte geben Sie Ihre berufliche Tätigkeit an.   - Hausarzt oder Hausärztin - Spezialisierte*r Facharzt oder Fachärztin - Medizinische*r Praxisassistent*in oder medizinische*r Praxiskoordinator*in - Pflegefachperson - Psychotherapeut*in - Psycholog*in - Physiotherapeut*in - Ernährungsberater*in - Ergotherapeut*in - Sozialarbeiter*in - Seelsorger*in - Freiwillige oder Ehrenamtliche (oder Ähnliche) - Andere: |
| Bitte geben Sie Ihre Anstellungsform an.   - Selbständig erwerbend oder in eigener AG tätig. - Angestellte*r - Andere: |
| Wie viele Stunden pro Woche arbeiten Sie? |
| Bitte geben Sie Ihr Arbeitspensum in % an. |
| Bitte geben Sie Ihre Berufserfahrung (ohne Ausbildung) in Jahren an. |
| Haben Sie eine Weiterbildung in Palliative Care, wenn ja welche? |

**Was ist Interprofessionalität?**Laut der WHO findet interprofessionelle Zusammenarbeit statt, wenn mehrere Mitarbeitende des Gesundheitswesens mit unterschiedlichem beruflichem Hintergrund mit Patienten und Patientinnen, Familien, Pflegefachpersonen und Institutionen zusammenarbeiten, um eine qualitativ hochwertige Versorgung in verschiedenen Settings des Gesundheitswesens zu gewährleisten.

**Einstiegsfrage**

|  | Stimme überhaupt nicht zu | Stimme  eher nicht zu | Stimme teilweise zu | Stimme voll und ganz zu |
| --- | --- | --- | --- | --- |
| Ich finde interprofessionelle Zusammenarbeit wichtig und sinnvoll. |  |  |  |  |

**Interprofessionelle Zusammenarbeit**

Bitte kreuzen Sie in den nachfolgenden Fragen, wie oft Sie mit den Berufsgruppen zusammengearbeitet haben.

| Mit welchen der folgenden Berufsgruppen haben Sie seit dem 01. August 2022 im Rahmen des SMPCD Pilot zusammengearbeitet? | Mind. einmal jeden Tag | Mind. einmal die Woche | Mind. einmal im Monat | Einige Male seit dem 1.8.2022 | Nie |
| --- | --- | --- | --- | --- | --- |
| Hausarzt oder Hausärztin |  |  |  |  |  |
| Spezialisierte*r Facharzt oder Fachärztin |  |  |  |  |  |
| Medizinische*r Praxisassistent*in oder medizinische*r Praxiskoordinator*in |  |  |  |  |  |
| Pflegefachperson |  |  |  |  |  |
| Psycholog*in |  |  |  |  |  |
| Psychotherapeut*in |  |  |  |  |  |
| Physiotherapeut*in |  |  |  |  |  |
| Ernährungsberater*in |  |  |  |  |  |
| Sozialarbeiter*in |  |  |  |  |  |
| Seelsorger*in |  |  |  |  |  |
| Freiwillige oder Ehrenamtliche (oder Ähnliche) |  |  |  |  |  |
| Andere: |  |  |  |  |  |

**Fragen zur interprofessionellen Zusammenarbeit** während des Pilotprojektes des spezialisierten mobilen Palliative Care Dienstes (SMPCD). Bitte schätzen Sie die interprofessionelle Zusammenarbeit zwischen der eigenen und den jeweils anderen Berufsgruppen ein, mit denen Sie **mindestens einmal pro Monat** zusammengearbeitet haben.

| 1. Meine Berufsgruppe und die andere Berufsgruppe sind sich bezüglich ihrer jeweiligen Verantwortlichkeit einig. | Stimme überhaupt nicht zu | Stimme eher nicht zu | Stimme teilweise zu | Stimme voll und ganz zu | Nicht relevant |
| --- | --- | --- | --- | --- | --- |
| Hausarzt oder Hausärztin |  |  |  |  |  |
| Spezialisierte*r Facharzt oder Fachärztin |  |  |  |  |  |
| Medizinische*r Praxisassistent*in oder medizinische*r Praxiskoordinator*in |  |  |  |  |  |
| Pflegefachperson |  |  |  |  |  |
| Psycholog*in |  |  |  |  |  |
| Psychotherapeut*in |  |  |  |  |  |
| Physiotherapeut*in |  |  |  |  |  |
| Ernährungsberater*in |  |  |  |  |  |
| Sozialarbeiter*in |  |  |  |  |  |
| Seelsorger*in |  |  |  |  |  |
| Freiwillige oder Ehrenamtliche (oder Ähnliche) |  |  |  |  |  |
| Andere: |  |  |  |  |  |

**Interprofessionelle Zusammenarbeit**

Bitte schätzen Sie die interprofessionelle Zusammenarbeit zwischen der eigenen und den jeweils anderen Berufsgruppen ein, mit denen Sie **mindestens einmal pro Monat** zusammengearbeitet haben.

|  | Stimme überhaupt nicht zu | Stimme eher nicht zu | Stimme teilweise zu | Stimme voll und ganz zu | Betrifft mich nicht |
| --- | --- | --- | --- | --- | --- |
| 2. Die andere Berufsgruppe ist in der Regel bereit, unsere Arbeit bei der Planung ihrer Arbeit zu berücksichtigen. |  |  |  |  |  |
| 3. Ich finde, die Behandlung und Betreuung der Patienten und Patientinnen wird von uns mit der anderen Berufsgruppe angemessen diskutiert. |  |  |  |  |  |
| 4. Die andere Berufsgruppe und meine Berufsgruppe haben ähnliche Vorstellungen, wie die Patienten und Patientinnen behandelt und betreut werden sollten. |  |  |  |  |  |
| 5. Die andere Berufsgruppe ist bereit, mit meiner Berufsgruppe über klinische Fragen zu diskutieren. |  |  |  |  |  |
| 6. Die andere Berufsgruppe ist bereit, sich auf die Art und Weise einzustellen, wie wir unsere Arbeit organisieren. |  |  |  |  |  |
| 7. Die andere Berufsgruppe ist bereit, sich auf neue Arbeitsweisen meiner Berufsgruppe einzustellen. |  |  |  |  |  |
| 8. Die andere Berufsgruppe fragt normalerweise nicht nach unserer Meinung. |  |  |  |  |  |
| 9. Die andere Berufsgruppe erkennt, wenn wir ihre Unterstützung benötigen. |  |  |  |  |  |
| 10. Wichtige Informationen werden von uns immer an die andere Berufsgruppe weitergegeben. |  |  |  |  |  |
| 11. Meinungsverschiedenheiten mit der anderen Berufsgruppe bleiben oft ungeklärt. |  |  |  |  |  |
| 12. Die andere Berufsgruppe ist der Meinung, ihre Arbeit sei wichtiger als unsere. |  |  |  |  |  |
| 13. Die andere Berufsgruppe ist bereit, ihre neuen Arbeitsweisen mit uns zu diskutieren. |  |  |  |  |  |

**Patientenverfügung, Vorsorgeauftrag, SMPCD Formulare & Erreichbarkeit von Hausärztinnen oder Hausärzten**

Im Rahmen der interprofessionellen Zusammenarbeit ist es hilfreich zu erfahren, welche Erfahrungen Sie mit Patientenverfügungen, Vorsorgeaufträgen, SMPCD Formularen und die Erreichbarkeit von Hausärztinnen oder Hausärzten gemacht haben. Die nachfolgenden Fragen decken diese vier Themen ab.

**Patientenverfügung**

|  | Stimme überhaupt nicht zu | Stimme eher nicht zu | Stimme teilweise zu | Stimme voll und ganz zu | Betrifft mich nicht |
| --- | --- | --- | --- | --- | --- |
| 14. Die Patientenverfügung ist ein notwendiges Element in der Palliative Care. |  |  |  |  |  |
| 15. Die Patientenverfügung war bei allen SMPCD involvierten Patienten und Patientinnen, mit denen ich bereits Kontakt hatte, ausgefüllt und besprochen. |  |  |  |  |  |
| 16. Ich weiss, wo die Patientenverfügung zu finden ist. |  |  |  |  |  |

**Vorsorgeauftrag**

|  | Stimme überhaupt nicht zu | Stimme eher nicht zu | Stimme teilweise zu | Stimme voll und ganz zu | Betrifft mich nicht |
| --- | --- | --- | --- | --- | --- |
| 17. Der Vorsorgeauftrag ist ein notwendiges Element in der spezialisierten Palliative Care. |  |  |  |  |  |
| 18. Der Vorsorgeauftrag war bei allen SMPCD involvierten Patienten und Patientinnen, mit denen ich bereits Kontakt hatte, vorhanden und besprochen. |  |  |  |  |  |

**Fachformulare**

|  | Stimme überhaupt nicht zu | Stimme eher nicht zu | Stimme teilweise zu | Stimme voll und ganz zu | Betrifft mich nicht |
| --- | --- | --- | --- | --- | --- |
| 19. Der palliative Betreuungsplan (PBP)... |  |  |  |  |  |
| ... ist ein notwendiges Dokument des SMPCD. |  |  |  |  |  |
| ... war bei allen SMPCD involvierten Patienten und Patientinnen, mit denen ich bereits Kontakt hatte, vollständig aufgefüllt und aktuell. |  |  |  |  |  |
| ... ist verständlich und unterstützt mich in der Patientenversorgung. |  |  |  |  |  |

|  | Stimme überhaupt nicht zu | Stimme eher nicht zu | Stimme teilweise zu | Stimme voll und ganz zu | Betrifft mich nicht |
| --- | --- | --- | --- | --- | --- |
| 20. Der medikamentöse Notfallplan... |  |  |  |  |  |
| ... ist ein notwendiges Dokument des SMPCD. |  |  |  |  |  |
| ... war bei allen SMPCD involvierten Patienten und Patientinnen, mit denen ich bereits Kontakt hatte, vollständig aufgefüllt und aktuell. |  |  |  |  |  |
| ... ist verständlich und unterstützt mich in der Patientenversorgung. |  |  |  |  |  |

|  | Stimme überhaupt nicht zu | Stimme eher nicht zu | Stimme teilweise zu | Stimme voll und ganz zu | Betrifft mich nicht |
| --- | --- | --- | --- | --- | --- |
| 21. Die Anleitung für Patienten und Patientinnen und deren Angehörige... |  |  |  |  |  |
| ... ist ein notwendiges Dokument des SMPCD. |  |  |  |  |  |
| ... war bei allen SMPCD involvierten Patienten und Patientinnen, mit denen ich bereits Kontakt hatte, vollständig aufgefüllt und aktuell. |  |  |  |  |  |
| ... ist verständlich und unterstützt mich in der Patientenversorgung. |  |  |  |  |  |

**Erreichbarkeit der Hausärzte und Hausärztinnen**

|  | Stimme überhaupt nicht zu | Stimme eher nicht zu | Stimme teilweise zu | Stimme voll und ganz zu | Betrifft mich nicht |
| --- | --- | --- | --- | --- | --- |
| 22. Der zuständige Hausarzt oder die zuständige Hausärztin ist bei Unklarheiten telefonisch erreichbar und antwortet zeitnahe. |  |  |  |  |  |
