# Supplementary material for: Interprofessional collaboration during a specialised mobile palliative care service pilot in the rural area of Lucerne
Source: PLoS One. 2024 Sep 18;19(9):e0308256. doi: 10.1371/journal.pone.0308256 (PMC11410264; doi:10.1371/journal.pone.0308256)
Supplement: S2 File — (DOCX) [file pone.0308256.s002.docx]

1. SMPCS standardised documents
   1. Guidance for patients and relatives

- 1. Palliative care plan
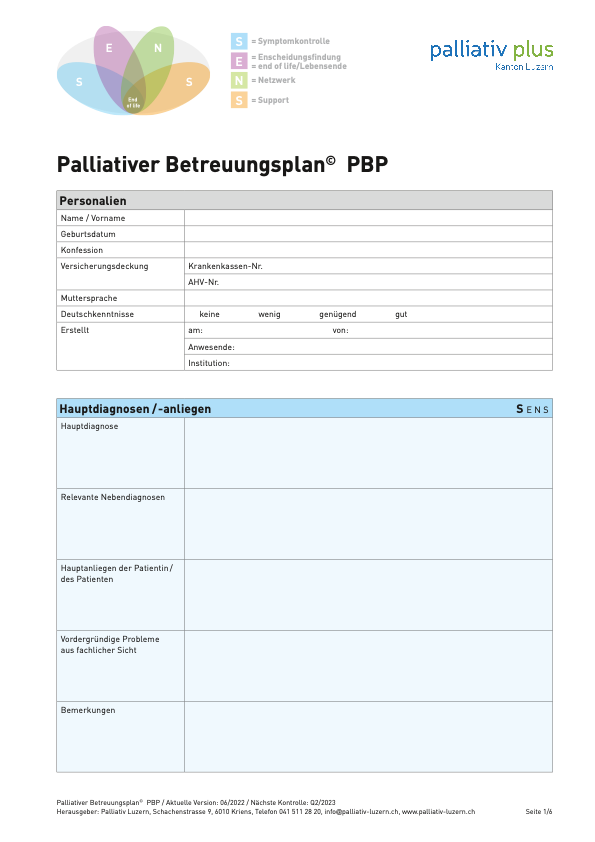

- 1. Medication emergency plan
