# Supplementary material for: Interprofessional collaboration during a specialised mobile palliative care service pilot in the rural area of Lucerne
Source: PLoS One. 2024 Sep 18;19(9):e0308256. doi: 10.1371/journal.pone.0308256 (PMC11410264; doi:10.1371/journal.pone.0308256)
Supplement: S3 File — (DOCX) [file pone.0308256.s003.docx]

1. Interview guide
   1. Interview guide nurses

| **Demographische Daten** |
| --- |
| - Wie alt sind Sie? - Welches Geschlecht haben Sie? - Was ist Ihre berufliche Tätigkeit? - Wo arbeiten Sie? - Wie ist Ihre Anstellungsform? (Selbständig erwerbend oder in eigener AG tätig, Angestelle*r) - Wie viele Stunden arbeiten Sie pro Woche? - Was ist Ihr aktuelles Arbeitspensum? - Wie viele Jahre Berufserfahrung haben Sie (ohne Ausbildung)? - Haben Sie eine Weiterbildung in Palliative Care, wenn ja welche? |
| **Austausch** |
| **Begriffserklärung**   - - Ist Ihnen der Begriff Interprofessionalität bekannt?   🡪 Wie würden Sie interprofessionelle Zusammenarbeit beschreiben?  🡪 Wie finden Sie interprofessionelle Arbeit?  🡪 Wenn nein, Erklärung des Begriffs^1^.  🡪 Wie finden Sie interprofessionelle Arbeit?  **Erlebte Interprofessionalität im Pilotprojekt SMPCD**   - - Wie haben Sie die interprofessionelle Zusammenarbeit im SMPCD Pilotprojekt seit dem 01. August erlebt?   - Mit welchen Berufsgruppen haben Sie in Ihrem Arbeitsalltag zu tun?   - Wie schwer ist es für Sie mit anderen Berufsgruppen in Kontakt zu kommen?   🡪 Wieso kommt es dazu, dass Sie zu gewissen Berufsgruppen weniger oder keinen Kontakt haben?   - - Wäre es Ihrer Meinung nach wichtig, den Kontakt zu den anderen Berufsgruppen zu haben, mit denen Sie nicht so viel oder gar nicht zu tun haben? Wieso?   - Falls mit allen Kontakten: Wie es für Sie mit allen Berufsgruppen im Kontakt zu sein?   🡪 Was für eine Rolle haben Sie dabei?  🡪 Wie erleben Sie den Kontakt zu anderen Berufsgruppen?  🡪 Wie managen Sie die Rolle, in welcher Sie mit allen Berufsgruppen in Kontakt sind?  🡪 Was ist Ihnen in dieser Position besonders wichtig?  **Förderliche und hinderliche Faktoren**   - Was braucht es Ihrer Meinung nach, für eine erfolgreiche interprofessionelle Zusammenarbeit? - Was sind Faktoren, die eine erfolgreiche interprofessionelle Zusammenarbeit hemmen können? |
| **Kommunikation** |
| - - Wenn Sie an den Austausch mit anderen Berufsgruppen denken, wie halten Sie diesen aufrecht?   🡪 Welche Kommunikationsmittel nutzen Sie dafür?   - - Was sind Ihrer Meinung nach förderliche Faktoren für eine gelungene Kommunikation?   - Was sind Ihrer Meinung nach hinderliche Faktoren für eine gelungene Kommunikation? |
| **Rollenverteilung** |
| - Wie fänden Sie es pro Fall eine Ansprechperson (sie Case Manager oder eine APN) zu haben, welcher den Kontakt zu allen Berufsgruppen hat?   🡪 Welchen Einfluss, hätte Ihrer Meinung nach, eine solche Rolle für die interdisziplinäre Zusammenarbeit? Wieso eher belastend oder wieso eher hilfreich?   - Wie gut kennen Sie, die Aufgaben von anderen Berufsgruppen?   🡪 Wie wichtig ist Ihrer Meinung nach zu Wissen, was die anderen Berufsgruppen für Aufgaben haben? |
| **Organisation** |
| - Wie schätzen Sie die Zeit, die Sie für die Bearbeitung der patientenrelevanten Dokumente erhalten, ein?   🡪 Können Sie diesen Aufwand verrechnen?  🡪 Wenn nein: Wieso nicht? Ist dies ein hinderlicher Faktor im Zusammenhang für die interprofessionelle Zusammenarbeit?   - Wie vertraut sind Ihnen die Fachformulare des SMPCD? (Darunter auch Notfallplan)   🡪 Wie nutzen Sie diese Dokumente?  🡪 Wie finden Sie diese Fachformulare?  🡪 Wie hilfreich sind Ihnen diese Fachformulare in Ihrem Arbeitsalltag?  🡪 Welchen Einfluss, haben Ihrer Meinung nach, die Fachformulare für die interprofessionelle Zusammenarbeit?   - Welchen Stellenwert hat für Sie die Patientenverfügung in der spezialisierten palliativen Pflege?   🡪 Wer trägt Ihrer Meinung nach, die Verantwortung darüber, dass die Patientenverfügung ausgefüllt und aktuell ist? Wieso?  🡪Falls eher geringer Stellenwert: Wieso?   - Wie schätzen Sie die Erreichbarkeit der anderen Berufsgruppen ein? - Kommen Sie stets zu Ihren gewünschten und benötigten Informationen?   🡪 Wenn nicht: Welche Schwierigkeiten herrschen?  🡪 Wenn ja: Was macht es aus, dass es so gut funktioniert? |
| **Abschlussfragen (falls nicht schon beantwortet)** |
| - - Wie viele SMPCD Patienten und Patientinnen betreuen Sie zurzeit oder haben Sie seit dem 01. August bereits betreut?   - Wie regelmässig haben Sie Kontakt zu SMPCD Patienten und Patientinnen?   - Wie wissen Sie oder wie erfahren Sie das Patienten und Patientinnen im SMPCD Projekt eingeschlossen sind? |
| **Abschluss** |
| - Gibt es Dinge, die Sie noch erwähnen möchten, zu denen Sie bis jetzt nicht gekommen sind?   🡪 Hiermit wäre das Interview fertig. Ich stoppe die Aufzeichnung und bedanke mich für Ihre Zeit. |

**^1^Defintion Interprofessionalität**Laut der WHO findet interprofessionelle Zusammenarbeit statt, wenn mehrere Mitarbeiter des Gesundheitswesens mit unterschiedlichem beruflichem Hintergrund mit Patienten und Patientinnen, Familien, Pflegefachpersonen und Institutionen zusammenarbeiten, um eine qualitativ hochwertige Versorgung in verschiedenen Settings des Gesundheitswesens zu gewährleisten.

- 1. Interview guide primary care physicians

| **Demographische Daten** |
| --- |
| - Wie alt sind Sie? - Welches Geschlecht haben Sie? - Was ist Ihre berufliche Tätigkeit? - Wo arbeiten Sie? - Wie ist Ihre Anstellungsform? (Selbständig erwerbend oder in eigener AG tätig, Angestelle*r) - Wie viele Stunden arbeiten Sie pro Woche? - Was ist Ihr aktuelles Arbeitspensum? - Wie viele Jahre Berufserfahrung haben Sie (ohne Ausbildung)? - Haben Sie eine Weiterbildung in Palliative Care, wenn ja welche? |
| **Austausch** |
| **Begriffserklärung**   - - Ist Ihnen der Begriff Interprofessionalität bekannt?   🡪 Wenn ja  🡪 Wie würden Sie interprofessionelle Zusammenarbeit beschreiben?  🡪 Wie finden Sie interprofessionelle Arbeit?  🡪 Wenn nein, Erklärung des Begriffs^1^.  🡪 Wie finden Sie interprofessionelle Arbeit?  **Erlebte Interprofessionalität im Pilotprojekt SMPCD**   - - Wie haben Sie die interprofessionelle Zusammenarbeit im SMPCD Pilotprojekt seit dem 01. August erlebt?   - Mit welchen Berufsgruppen haben Sie in Ihrem Arbeitsalltag zu tun?   - Wie schwer ist es für Sie mit anderen Berufsgruppen in Kontakt zu kommen?   🡪 Wieso kommt es dazu, dass Sie zu gewissen Berufsgruppen weniger oder keinen Kontakt haben?   - - Machen Sie Hausbesuche? Welchen Einfluss haben Ihrer Meinung nach Hausbesuchen auf die interprofessionelle Zusammenarbeit?   **Förderliche und hinderliche Faktoren**   - Was braucht es Ihrer Meinung nach, für eine erfolgreiche interprofessionelle Zusammenarbeit? - Was sind Faktoren, die eine erfolgreiche interprofessionelle Zusammenarbeit hemmen können? |
| **Kommunikation** |
| - - Wenn Sie an den Austausch mit anderen Berufsgruppen denken, wie halten Sie diesen aufrecht?   🡪 Welche Kommunikationsmittel nutzen Sie dafür?  🡪 Was läuft dabei reibungslos und wieso?  🡪 Was ist dabei problematisch und wieso?   - - Was sind Ihrer Meinung nach förderliche Faktoren für eine gelungene Kommunikation?   - Was sind Ihrer Meinung nach hinderliche Faktoren für eine gelungene Kommunikation? |
| **Rollenverteilung** |
| - Der SMPCD wurde den Hausärzten und Hausärztinnen von der Spitex aus vorgestellt, dafür haben sich die Pflegefachfrauen mit den Hausärzten und Hausärztinnen getroffen, wie war Ihre Erfahrung dazu?   🡪 Hat dieser persönliche Kontakt die interprofessionelle Zusammenarbeit gefördert?   - Wie gut kennen Sie, die Aufgaben von anderen Berufsgruppen?   🡪 Wie wichtig ist Ihrer Meinung nach zu Wissen, was die anderen Berufsgruppen für Aufgaben haben?   - Wie fänden Sie es pro Fall eine Ansprechperson (sie Case Manager oder eine APN) zu haben, welcher den Kontakt zu allen Berufsgruppen hat?   🡪 Welchen Einfluss, hätte Ihrer Meinung nach, eine solche Rolle für die interdisziplinäre Zusammenarbeit? Wieso eher belastend oder wieso eher hilfreich? |
| **Organisation** |
| - Wie schätzen Sie die Zeit, die Sie für die Bearbeitung der patientenrelevanten Dokumente erhalten, ein?   🡪 Können Sie diesen Aufwand verrechnen?  🡪 Wenn nein: Wieso nicht? Ist dies ein hinderlicher Faktor im Zusammenhang für die interprofessionelle Zusammenarbeit?   - Wie vertraut sind Ihnen die Fachformulare des SMPCD? (Darunter auch Notfallplan)   🡪 Wie nutzen Sie diese Dokumente?  🡪 Wie finden Sie diese Fachformulare?  🡪 Wie hilfreich sind Ihnen diese Fachformulare in Ihrem Arbeitsalltag?  🡪 Welchen Einfluss, haben Ihrer Meinung nach, die Fachformulare für die interprofessionelle Zusammenarbeit?   - Welchen Stellenwert hat für Sie die Patientenverfügung in der spezialisierten palliativen Pflege?   🡪 Wer trägt Ihrer Meinung nach, vdie Verantwortung darüber, dass die Patientenverfügung ausgefüllt und aktuell ist? Wieso?  🡪Falls eher geringer Stellenwert: Wieso?   - Wie schätzen Sie die Erreichbarkeit der anderen Berufsgruppen ein? - Kommen Sie stets zu Ihren gewünschten und benötigten Informationen?   🡪 Wenn nicht: Welche Schwierigkeiten herrschen?  🡪 Wenn ja: Was macht es aus, dass es so gut funktioniert? |
| **Abschlussfragen (falls nicht schon beantwortet)** |
| - - Wie viele SMPCD Patienten und Patientinnen betreuen Sie zurzeit oder haben Sie seit dem 01. August bereits betreut?   - Wie regelmässig haben Sie Kontakt zu SMPCD Patienten und Patientinnen?   - Wie wissen Sie oder wie erfahren Sie das Patienten und Patientinnen im SMPCD Projekt eingeschlossen sind? |
| **Abschluss** |
| - Gibt es Dinge, die Sie noch erwähnen möchten, zu denen Sie bis jetzt nicht gekommen sind?   🡪 Hiermit wäre das Interview fertig. Ich stoppe die Aufzeichnung und bedanke mich für Ihre Zeit. |

**^1^Defintion Interprofessionalität**Laut der WHO findet interprofessionelle Zusammenarbeit statt, wenn mehrere Mitarbeiter des Gesundheitswesens mit unterschiedlichem beruflichem Hintergrund mit Patienten und Patientinnen, Familien, Pflegefachpersonen und Institutionen zusammenarbeiten, um eine qualitativ hochwertige Versorgung in verschiedenen Settings des Gesundheitswesens zu gewährleisten.
