# Supplementary material for: Interprofessional collaboration during a specialised mobile palliative care service pilot in the rural area of Lucerne
Source: PLoS One. 2024 Sep 18;19(9):e0308256. doi: 10.1371/journal.pone.0308256 (PMC11410264; doi:10.1371/journal.pone.0308256)
Supplement: Attachment — Submitted filename: Response to Reviewers.docx [file pone.0308256.s004.docx]

**Responses to Reviewers**

We would like to express our sincere gratitude for your review and constructive feedback. Enclosed, you will find detailed and precise responses to your suggestions. Direct revisions can be found in the document titled “revised manuscript with tracked changes”. We appreciate your cooperation throughout this process.

**Editor comments**

**Comment 1 (C1): There are too many abbreviations, some are used without correct introduction.**

We appreciate your feedback, and abbreviations have been reduced accordingly. You will find the adjustments in the revised manuscript “with tracked changes”.

**C2: One should discuss the reasons why the interviewer proceeded “without further training in conducting interviews” (page 5). This weakness would also merit a brief discussion.**

Following the COREQ guidelines, the qualifications of the interviewer were duly recorded. The term "without further training" was utilized to denote the completion of specialized training, notwithstanding the interviewer's limited practical experience. The interviewer acquired knowledge in qualitative methodologies and data collection during her undergraduate and postgraduate studies. The second author possesses a doctoral degree along with supplementary training in qualitative analysis. In the reliability testing, the second author served as the second rater and supervised the data collection and analysis processes to ensure methodological rigor. These credentials were duly recognized as potential constraints within the "strengths and limitations" delineation in Section 4.

**Section 4.4:** The person conducting the interview had basic knowledge in interviewing, which may have influenced the results.

**Feedback Reviewer #1**

On behalf of our research team, I would like to thank you Prof. Dr. Jan Gärtner for the time you have invested in reviewing this work.

**C3: You may consider adding some information about the evidence base of specialist pallative care.**

We would like to thank you very much for drawing our attention to this valuable study about the topic specialized palliative care, which we have gladly implemented in our work.

**Feedback Reviewer #2**

On behalf of our research team, I would like to thank you for the time you have invested in reviewing our work. Your constructive comments have enabled us to further refine and establish our work, for which we are grateful. In the text below, you will find the answer to your suggestions with the references to the corrections in the manuscript.

**C4: Authors should precisely state a research question.**

The research question has now been added.

**Section 1:** The following research question was addressed: what were FaBs of interprofessional collaboration during the SMPCS pilot project in a rural part of Switzerland?

**C5: Better describe the pilot project with major points that will enable the readers to understand how the mobile team was formed, which professionals are part of this team and which model of care was used.**

Thank you for this important comment. These issues have now been addressed as follows:

**Section 2.1:** The primary group within the specialized palliative care service consisted of qualified nurses with further training in specialized palliative care and primary care physicians. The nursing professionals were employed within existing home care organizations in the rural regions of Lucerne., The pilot project was structured in a highly dynamic manner whereas additional professional groups were incorporated based on the patients’ specific needs. This approach facilitated individualized, specialized, and patient-centered care for each palliative care patient. There were no predefined individuals assigned to the pilot project from the outset; rather, they were integrated into the pilot project team as needed throughout its course.

**C6: Authors should reassure the reviewers about the respect of the anonymity of participants or provide less identifying details.**

The study was conducted in accordance with Swiss ethical standards and regulations. The interviews were anonymized to ensure that no inferences could be drawn about individual participants. All potentially identifying statements, such as those regarding localities, names, or businesses, were anonymized, making these inaccessible to the public. These issues have now been further addressed as follows:

**Section 2.3.1:** Participants provided informed consent based on the Swiss federal law on data protection and were informed that they would remain anonymous. The anonymization procedure was adopted from Kuckartz & Rädiker (2022) and applied according to their guidelines (11). As no patient data were involved, an ethics committee application was not required under Swiss law of data protection, specifically under the Swiss federal act on research involving human beings (11,12).

**C7: Mention inclusion and exclusion criteria of participants and, given the aim to investigate interprofessional collaboration, precisely mention what were the professions of the “professional groups (PG) “. Were all part of the palliative care specialised team? Were they part of the home care primary nurses, GPs or others? It needs a clear description of the population interviewed.**

Thank you for this important comment. These issues have now been addressed as follows:

**Section 2.1:** This study defines professional groups as a collection of diverse professionals who have direct or indirect interactions with patients receiving palliative care. The primary group within the specialized palliative care consisted of qualified nurses with further training in specialized palliative care and primary care physicians. The nursing professionals were employed within existing home care organizations in the rural regions of Lucerne.

**Section 2.2.1:** Only qualified nurses and PCPs, aged 18 and above, who had direct or indirect contact with patients at least once in the context of the SMPCS pilot project were approached and included for the interviews.

**Section 2.2.2:** Only individuals aged 18 and older who had direct or indirect contact with patients were included. … All professionals were categorized into one of the following seven professional groups: primary care physicians, nurses, psychologists, psychotherapists, medical practice assistants or specialized medical assistants, occupational therapists, nutritionists, social workers, pastoral care providers, and volunteers.

**C8: Why were only professionals of the specialised mobile team invited to participate in the quantitative part of the study?**

Our study focused on investigating the specific facilitators and barriers to collaboration among professional groups involved in direct or indirect patient care within the context of the specialized mobile palliative care pilot.

We had addressed this in section 1 as follows:

**Section 1.:** Such shortcomings in professional collaboration can lead to the inadequate treatment of patients. To optimize interprofessional collaboration, its specific facilitators and barriers must be identified among professional groups involved in direct or indirect patient care within the context of the specialized mobile palliative care pilot. Especially during a pilot, interprofessional collaboration is still forming and establishing; moreover, various professional groups often come together for the first time, making interprofessional collaboration even more challenging.

**C9: Given that Reeves’ theory was used to analyse the data, a brief explanation of why this theory was chosen and why only two factors of this theory were used in a separate set of interviews should be provided.**

Thank you for pointing this out. We have now clarified this in two sections:

**Section 2.4.1:** We aimed to construct our work upon pre-existing, evidence-based knowledge, utilizing Reeves' theoretical framework, which delved into interprofessional collaboration and delineated four primary categories within its construct, namely: relational, procedural, organizational, and contextual.

**Section 2.4.1:** All six interviews included the application of these four categories of Reeves' theoretical framework. In terms of content, the interviews with nursing staff were fully consistent with the aforementioned categories (see Appendix 8). In contrast, the interviews with primary care providers only matched two of the four defined categories, namely: relational and procedural.

**C10: Why was content analysis chosen as a method to analyse interviews? How does content analysis link with a coding framework deductively chosen?**

Content analysis was selected as the method for analyzing interviews due to its systematic and objective approach. It is particularly suitable for this present study as a method to explore qualitative data in a structured manner, providing a framework for organizing and interpreting the information gathered during interviews.

This study primarily adopted a deductive approach as its main strategy for three reasons.

**Section 2.4.1:** (I) A deductive approach was chosen because the utilization of the theoretical framework provides a solid foundation for data analysis derived from relevant theories and existing knowledge. (II) A deductive approach can offer the opportunity to focus the analysis on specific aspects that are relevant to the research question**.** (III) This approach facilitates the systematic categorization of the interview content according to the predetermined framework and its concepts, enhancing the reliability and validity of the analysis.

**C11: The results section is divided in paragraphs with titles. How are the titles linked to the codes that emerged from the content analysis? The paragraphs are mostly descriptive of the collected data. There doesn’t appear to have a link with a coding, and a higher conceptualisation of data is needed, and a clear link to Reeves’s four factors should emerge.**

Reeves' theory provided a framework for the coding manual. Utilizing this coding manual, Reeves' interviews were deductively analyzed. The rationale behind this approach was to apply existing knowledge and categorize the interviews based on the predetermined titles from Reeves' theory.

The titles in Section 4 were derived from the content analysis of Reeves' theory and show therefore a clear link to the content analysis. Reeves' theory is omnipresent; although not explicitly mentioned, all content is founded upon this theory.

In order to highlight the connection between Reeve's theory and our analysis, we made the following changes:

**Section 3.1:** The results section comprises qualitative and quantitative components. The qualitative section highlights seven key findings from the analysis, relying on two primary factors from Reeves’ theory: processual and relational. Processual factors, detailed in chapters 3.1.2 to 3.1.4, encompass elements like time, routines, technologies, and situational complexity, influencing interprofessional collaboration. Similarly, relational factors, discussed in chapters 3.1.5 to 3.1.7, include socialization, team dynamics, roles, and professional authority, contributing to interprofessional collaboration.

**C12: The number of participants in the survey is too low (17 participants) to contribute substantially to the understanding of the qualitative part nor allows to derive statistically substantiated data.**

We acknowledge that our limited sample size hampers statistical calculations and inferential statistics. Consequently, we have only conducted descriptive data analysis as described in section 2.4.2. Furthermore, this limitation was highlighted in the section 4.4 “strengths and limitations”.

We nevertheless wish to emphasize the importance of including additional professional groups. The comprehensive understanding of the pilot can only be achieved through the perspectives and opinions of further involved professional groups. We have now clarified this in the following section:

**Section 2.1:** With this method, we aimed to incorporate the perspectives of other involved professional groups, in addition to nurses and primary care physicians.

**C13: Was the qualitative part conducted before or after the survey? Why was this approach chosen?**

Both parts were conducted at the same time. We chose this approach to incorporate additional opinions and perspectives from other professional groups into the study. We have now clarified this in one section:

**Section 2.1:** A mixed-method approach was chosen to collect qualitative and quantitative data in parallel, aiming to gain a deeper understanding of the facilitators and barriers of the pilot. With this method, we aimed to incorporate the perspectives of other involved professional groups, in addition to nurses and primary care physicians.

**C14: Authors state that the results section for the qualitive part shows “relevant results”. Do they mean main themes or domains?**

The statement referring to relevant results refer to the identified themes of the qualitative component of this study. These were further elaborated upon in sections 3.1.1 to 3.1.7 and are as follows: Evaluation of interprofessional collaboration and professionals involved, standardised documents in specialised palliative care, time factor in interdisciplinary teams, team roles in interdisciplinary teams, care coordination in specialised palliative care, knowledge and experience in specialised palliative care, and exchange and communication in interdisciplinary teams.

**C15: The discussion section presents the facilitators and barriers of IPC. Their identification and analysis were stated as the aim of the research, for such reason should be considered as the results of the study.**

The results section presents the findings of the qualitative and quantitative data collection, categorized into these two areas to provide a clear structure. The discussion section interprets these results, subdividing them into facilitators and barriers. The absence of headings and the differentiation between facilitators and barriers in the results section does not negate their presence therein. Furthermore, while the results section aims for accuracy and concise presentation, the discussion section allows for more interpretative and analytical freedom. Additionally, this is particularly significant as not every result may be essential for practical application, yet the discussion can contribute to greater clarity on this matter.

However, we have now added an overview for both sections (Facilitators and Barriers) in chapter 4.2 and 4.3 to highlight the main findings before delving into more detail in the discussion’s subsections.

**C16: Data transcripts and coding book should not be provided for publication and interview grid and all study material presented in German should be selected and that relevant for publication should be translated into English. Quotes should be selected to represent the variety of opinions and translated into English.**

The entire material has been uploaded to ensure transparency. The interview grid and all study materials in German have now been removed from the “revised manuscript with track changes”. The quotes referenced in the manuscript are provided in their original form and had been additionally translated in Appendix 3 and 4.

**C17: The paper needs more work in its overall structure and the quality of academic English needs improvement.**

Thank you for your feedback. We have revised the paper in terms of its structure and enhanced the quality of academic English. Additionally, by reducing the use of abbreviations, we aimed to improve readability. After incorporating the revisions and adjustments prompted by the reviewers' feedback, the second author, proficient in bilingual English, reviewed the language usage.
