# Supplementary material for: Interprofessional collaboration during a specialised mobile palliative care service pilot in the rural area of Lucerne
Source: PLoS One. 2024 Sep 18;19(9):e0308256. doi: 10.1371/journal.pone.0308256 (PMC11410264; doi:10.1371/journal.pone.0308256)
Supplement: Attachment — Submitted filename: Response to Academic Editor.docx [file pone.0308256.s005.docx]

**Response to Editor**

We would like to express our sincere gratitude for your feedback. Below are detailed responses to your comments. The direct revisions based on the suggestions made in the last email dated June 6, 2024, are in the document titled “Revised Manuscript with Tracked Changes”. We appreciate your cooperation throughout this process.

Your suggestions to & / -ise / -ize / advanced have been corrected. Please note that in addition to the corrections, text reductions have been made throughout the manuscript.

**Editor comments**

**Comment 1: Please review your reference list to ensure that it is complete and correct.**

Citations 11 and 31 (see current revised manuscript) were added to the manuscript in the first revision based on the input of two reviewers about specialised palliative care and data protection. All 35 citations are listed in full in the current revised manuscript. No citations were omitted.

[12] Schweizerische Eidgenossenschaft. Bundesrecht: Bundesgesetz über die Forschung am Menschen (Humanforschungsgesetz, HFG) [Internet]. 2011 [cited 2024 Apr 13]. Available from: https://www.fedlex.admin.ch/eli/cc/2013/617/de.

[31] Gaertner J, Siemens W, Meerpohl JJ, Antes G, Meffert C, Xander C, et al. Effect of specialist palliative care services on quality of life in adults with advanced incurable illness in hospital, hospice, or community settings: systematic review and meta-analysis. BMJ. 2017 Jul 4;j2925.

**Comment 2: Why was there no calculation for the sample size (2.3.2 Quantitative data collection)?**

The main part of the results consists of the qualitative part, supplemented by the quantitative part; therefore, a sample size calculation was not planned due to the descriptive approach. Since no further testing and calculations were conducted and only a small population was requested, we limited it to a descriptive presentation without sample size determination.

**Section 2.1:** The use of mixed methods provided the opportunity to gain a deeper understanding of the interprofessional collaboration’s FaBs through interviews and to include the opinions of further involved professional groups via a questionnaire.

Furthermore, it should be mentioned that, for example, all nurses involved in the pilot phase participated in the survey, making it an almost complete data collection rather than a sample.

**Section 3.2:** All interviewed nurses participated in the questionnaire, and two out of three PCPs interviewed.

We have now clarified this in **section** **2.3.2**: Non-response was not addressed, item weighting was not conducted and a sample size calculation was not performed, due to the goal of describing the small group of involved professionals.

**Comment 3: Why differences in interviews with physicians and nurses (2.3.1 Qualitative data collection)?**

Because different professional groups have varying proximity to specialized palliative care patients and carry out different tasks and responsibilities, we supplemented the questionnaire with profession-specific questions to capture these distinct perspectives. However, the main body of questions remains identical across both groups to ensure comparability and to explore common themes in specialized palliative care provision from multiple professional angles.
